# Unsupervised Home Exercises Versus Formal Physical Therapy After Primary Total Hip Arthroplasty: A Systematic Review

**DOI:** 10.7759/cureus.29322

**Published:** 2022-09-19

**Authors:** Yash P Chaudhry, Hunter Hayes, Zachary Wells, Efstratios Papadelis, Alfonso Arevalo, Timothy Horan, Harpal S Khanuja, Carl Deirmengian

**Affiliations:** 1 Orthopaedic Surgery, Philadelphia College of Osteopathic Medicine, Philadelphia, USA; 2 Orthopaedics, Philadelphia College of Osteopathic Medicine, Philadelphia, USA; 3 Orthopaedic Surgery, Scripps Clinic, San Diego, USA; 4 Orthopaedic Surgery, Johns Hopkins University School of Medicine, Baltimore, USA; 5 Orthopaedic Surgery, The Rothman Orthopaedic Institute, Philadelphia, USA; 6 Orthopaedic Surgery, Thomas Jefferson University, Philadelphia, USA

**Keywords:** postoperative, rehabilitation, exercise, physical therapy, total hip arthroplasty

## Abstract

Historically, postoperative exercise and physical therapy (PT) have been viewed as crucial to a successful outcome following primary total hip arthroplasty (THA). This systematic review and meta-analysis aimed to assess differences in both short- and long-term objective and self-reported measures between primary THA patients with formal supervised physical therapy versus unsupervised home exercises after discharge. A search was conducted of six electronic databases from inception to December 14, 2020, for randomized controlled trials (RCTs) comparing changes from baseline in lower extremity strength (LES), aerobic capacity, and self-reported physical function and quality of life (QoL) between supervised and unsupervised physical therapy/exercise regimens following primary THA. Outcomes were separated into short-term (<6 months from surgery, closest to 3 months) and long-term (≥6 months from surgery, closest to 12 months) measures. Meta-analyses were performed when possible and reported in standardized mean differences (SMDs) with 95% confidence intervals (CI). Seven studies (N=398) were included for review. No significant differences were observed with regard to lower extremity strength (p=0.85), aerobic capacity (p=0.98), or short-term quality of life scores (p=0.18). Although patients in supervised physical therapy demonstrated improved short-term self-reported outcomes compared to those performing unsupervised exercises, this was represented by a small effect size (SMD 0.23 [95% CI, 0.02-0.44]; p=0.04). No differences were observed between groups regarding long-term lower extremity strength (p=0.24), physical outcome scores (p=0.37), or quality of life (p=0.14). The routine use of supervised physical therapy may not provide any clinically significant benefit over unsupervised exercises following primary THA. These results suggest that providers should reconsider the routine use of supervised physical therapy after discharge.

## Introduction and background

Formal physical therapy (PT) after total joint arthroplasty (TJA) is commonly recommended and is often thought to be indispensable to favorable patient outcomes [1,2]. In contrast to the modern era, these surgeries were performed on older, less active patients with more severe diseases and deformities [3]. These earlier surgeries were more extensive, and weight bearing was often limited postoperatively [4,5]. Modern total hip arthroplasty (THA), however, is characterized by a younger, more active patient population along with dramatic advances in pain control and rapid recovery [4,5]. Despite the changing landscape, formal PT has maintained its status amongst providers and patients as an integral component to improving outcomes [2,6].

Several studies over the past few years have demonstrated that formal PT, whether inpatient or outpatient, may not have any benefit over home-based unsupervised exercise programs [7-9]. While this has led to a shift away from formal PT utilization by some, there are no guidelines to assist in determining which patients may benefit [7]. Reducing the routine use of formal PT to only those patients for whom it is warranted may offer several benefits. The responsibility of copays and transportation is often placed on the patient and, for many, can be a significant burden [10,11]. Additionally, with the increased interest in alternative healthcare models, the focus has shifted to finding methods to reduce episode-of-care costs [12,13]. The costs associated with formal PT after discharge are not trivial, comprising up to 8% of total costs for TJA episodes of care [9].

The goal of this review was to assess the existing literature comparing outcomes of primary THA patients undergoing formal supervised PT to those with unsupervised home exercise programs through a systematic review and meta-analysis of randomized controlled trials (RCTs). To avoid intervention bias towards supervised home programs, we chose to only include studies that explicitly described unsupervised home exercise regimens. The primary aim was to assess changes in lower extremity strength (LES), aerobic capacity, and patient-reported physical outcome and quality of life (QoL) scores at zero to six months and six months to one year.

## Review

This systematic review and meta-analysis were conducted in accordance with the PRISMA (Preferred Reporting Items for Systematic Reviews and Meta-Analyses) guidelines and were registered in the PROSPERO International Prospective Register of Systematic Reviews (PROSPERO Identifier CRD42021228071).

Inclusion criteria

This review considered all English-language RCTs that compared objective measures and patient-reported outcomes (PROs) from patients with formal postoperative PT or supervised exercise programs to those with unsupervised home exercise interventions (defined as an explicitly stated form of home exercise program to be performed without direct supervision of a health professional). This also included written exercise instructions, video programs demonstrating exercises, or phone applications containing directions for exercises). The time period of interventions was limited to the period between discharge from hospitalization and six months postoperatively. Studies involving comparisons between only supervised cohorts, unsupervised cohorts, or without clear delineation of what each exercise intervention consists of were excluded, as were those involving preoperative exercise programs as the primary intervention.

Search strategy and study screening

With the assistance of an informationist, an electronic search was conducted of all published literature from database inception to December 14, 2020 from the following databases: PubMed, EMBASE, Web of Science, Scopus, Cochrane Library, and ClinicalTrials.gov. MeSH and Emtree terms were used alongside free text to enhance search sensitivity. Studies were screened based on titles and abstracts initially, with relevant studies subjected to full-text review. All screening was performed independently by two authors (YPC and HH). All disagreements were resolved through discussion, with input from the senior author (CAD) on an as-needed basis.

Quality appraisal

The Cochrane Risk of Bias Tool 2.0 was utilized to assess the five domains of potential bias: randomization process, deviations from intended intervention, missing outcome data, outcome measurement, and selection of reported results. The result for each domain was assigned risk scores of "low," "some concern," or "high." A risk of bias assessment was made for each outcome measurement. The GRADE (Grading of Recommendations Assessment, Development, and Evaluation) system was utilized to appraise the quality of evidence included in this meta-analysis to ensure the reliability of its results.

Data extraction and statistical analysis

Data extraction was performed manually by three reviewers (YPC, HH, and ZW). Extracted descriptive variables included journal, year, and country of publication, number of cases, age, gender, body mass index, inclusion criteria, follow-up, type of intervention, time from discharge to intervention initiation, and intervention length. The outcomes of interest in this study changed from baseline data in LES (measured with a timed up-and-go test (TUG), sit-to-stand test, or hip abduction strength as measured by a dynamometer), aerobic capacity, and patient-reported physical function and QoL. Each outcome measure was divided into short-term recovery (<6 months from surgery; if multiple time points were observed <6 months from surgery, the closest one to the three-month postoperative point was chosen) and long-term recovery (≥6 months from surgery; if multiple time points were observed ≥6 months from surgery, the closest one to the one-year postoperative point was chosen) windows. Changes from baseline values were collected in the form of a mean and standard deviation (SD). When not available for change from baseline scores, they were imputed using previously established methods from the Cochrane Handbook for Systematic Reviews of Interventions [14]. For studies involving more than one outcome measure for each of the above categories, only one outcome measure was included. Outcome measures were pooled for meta-analysis if there were at least three studies with reported results. All the outcomes in this study consisted of continuous variables. Effect sizes were assessed using random effect models to calculate standardized mean differences (SMD) and 95% confidence intervals (CI). Heterogeneity was tested using the I2 statistic. All meta-analysis calculations and subsequent forest plots were generated using Review Manager Software Version 5.4.1 (Copenhagen: The Nordic Cochrane Center, The Cochrane Collaboration, 2020).

Characteristics of included studies

**Figure 1:**
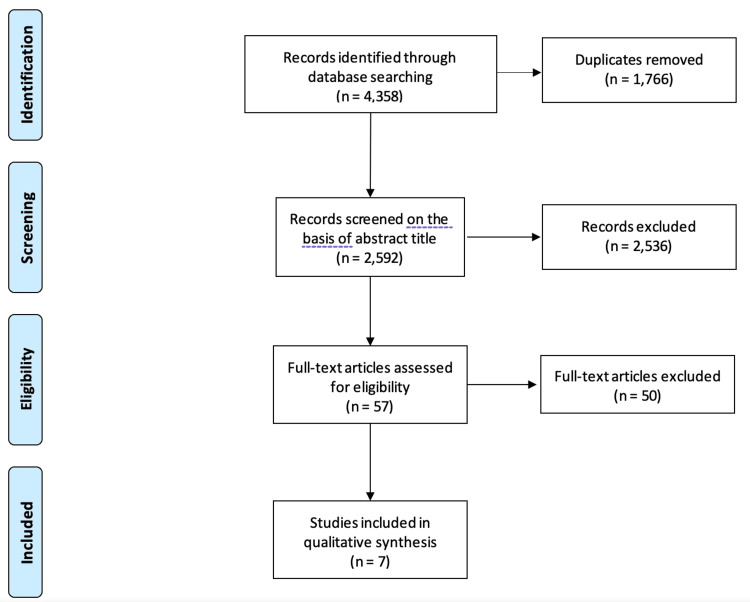
Preferred Reporting Items for Systematic Reviews and Meta-Analyses (PRISMA) flow chart.

A total of 4,358 citations were identified (Figure 1). After removing 1,766 duplicate citations, a total of 2,592 studies were assessed for eligibility based on title and abstract. Fifty-seven studies were eligible for full-text review. Ultimately, seven studies (n=398 cases) were included in this review.

A descriptive summary of the included studies can be found in Tables 1-2. All studies were performed within the last ten years, except for one conducted in 2008 [15]. Three of the seven studies began their intervention programs upon discharge; two in the first week after surgery, one six weeks after, and one program at 12 weeks postoperatively. The mean program length was 8.0 ± 2.8 weeks. Six studies reported outcomes in the short-term period, and five reported outcomes in the long-term period.

**Table 1:** Study characteristics of seven randomized controlled trials of unsupervised vs. supervised exercise regimens following primary total hip arthroplasty. BMI: body mass index; NR: not reported; THA: total hip arthroplasty; OA: osteoarthritis; HOOS: Hip disability and Osteoarthritis and Outcomes Score; ADL: activities of daily living. ^a^Median age – no mean age reported. ^b^Mean % female gender across all included studies. ^c^Mean BMI of studies with available data.

References	Country	N	Mean Age (years)	% Female	Mean BMI (kg/m^2^)	Inclusion/exclusion
Austin et al. [7]	USA	108	62	44	29	Inclusion: age 18-80 years, primary unilateral THA for OA. Exclusion: inflammatory or post-traumatic arthritis, history of septic arthritis, revision or conversion THA, patients requiring discharge to skilled facility.
Beaupre et al. [8]	Canada	21	53	52	NR	Inclusion: age <65 years, primary unilateral THA with lateral approach. Exclusion: history of developmental dysplasia of the hip.
Coulter et al. [16]	Australia	95	64^a^	58	NR	Inclusion: age > 18 years, primary elective THA, patient lives locally. Exclusion: metastatic disease, pathologic fractures, infection, acute trauma, revision THA, inability to provide informed consent, UCLA scale < 2 preoperatively, unable to bear weight postoperatively, requiring inpatient rehabilitation postoperatively.
Galea et al. [15]	Australia	23	68	30	29	Inclusion: primary THA for OA, ability to walk 45 minutes independently with mobility aid, independence in sit to stand transfer, adequately comprehends written/verbal instructions. Exclusion: uncontrolled systemic disease, pre-existing neurologic/other orthopedic condition affecting walking, more than four weeks physiotherapy postoperatively, revision surgery or significant postoperative complications
Mikkelsen et al. [17]	Denmark	62	65	42	29	Inclusion: primary unilateral THA for OA, preoperative HOOS ADL < 67, age > 18 years, live within 30 km from hospital, willing to participate. Exclusion: BMI > 35, pre-planned supervised rehabilitation, pre-planned contralateral THA within six months, inability to speak or read Danish, mental or physical conditions impeding intervention
Monaghan et al. [18]	Ireland	63	68	32	27	Inclusion: primary THA for OA, age > 50 years, able to read/understand English, willing to participate. Exclusion: medical instability, underlying terminal disease, suspicion of infection following THA
Okoro et al. [19]	United Kingdom	26	64	58	NR	Inclusion: unilateral THA for OA via posterior approach with 26/28/32 mm femoral head, joint affected is only arthritic joint, no evidence of inflammatory arthropathy. Exclusion: dementia, neurological impairment, cancer or other muscle wasting illness, unstable chronic or terminal illness, any co-morbid disease that contraindicates resistance training
Total		398	64	46^b^	29^c^	

**Table 2:** Study outcomes of seven randomized controlled trials of unsupervised vs. supervised exercise regimens following primary total hip arthroplasty. C: control; I: intervention; PT: physical therapy; HHS: Harris Hip Score; WOMAC: Western Ontario and McMaster’s Universities Osteoarthritis Index; SF: Short Form Survey; 6MWT: 6 Minute Walk Test; TUG: Timed Up and Go; STS: Sit to Stand; HOOS: Hip Disability Osteoarthritis and Outcomes Score; VAS: Visual Analogue Scale; DEXA: dual-energy x-ray absorptiometry. ^a^Mean program length. ^b^Isometric muscle strength and gait speed also checked at four weeks postoperatively.

References	Cohorts	Time from discharge to start	Program length (weeks)	Outcomes assessed	Follow-Up	Findings
Austin et al. [7]	C: 10 weeks unsupervised home exercise based on manual. I: 2 weeks in-home PT followed by 8 weeks outpatient PT 2-3x per week.	Upon discharge	10	HHS, WOMAC, SF-36 Physical and Mental	1 month, 6-12 months	No significant difference in any of measured outcomes
Beaupre et al. [8]	C: home exercise instructions for 4-6 weeks. I: outpatient rehabilitation 2x per week for 3 months.	6 weeks postoperatively	12	WOMAC, SF-36, 6MWT, gait analysis	4 months, 12 months	No significant difference in any of measured outcomes
Coulter et al. [16]	C: continue exercises from hospital at home, gradually increasing number of repetitions. I: supervised program 1x per week involving circuit exercises for 4 weeks.	Upon discharge	4	WOMAC, SF-36, TUG, UCLA activity index	5 weeks, 12 weeks, 26 weeks	No significant difference in any of measured outcomes
Galea et al. [15]	C: illustrated guide of prescribed home exercises. I: 45 minute sessions 2x per week in supervised rehabilitation center-based program.	First week after surgery	8	TUG, stair climb performance, 6MWT, WOMAC	8 weeks	C: with faster TUG. No significant difference in other measures.
Mikkelsen et al. [17]	C: home-based exercises done 7x per week. I: home-based exercises done 5x per week with additional supervised resistance training sessions 2x per week.	First week after surgery	10	Leg extension power, isometric hip muscle strength, STS, stair climb, 20 minute walking speed, HOOS	6 months^b^	I: with larger increase in maximal walking speed and stair climb performance. No significant difference in other measures
Monaghan et al. [18]	C: postoperative home exercise booklet, advised to walk daily with crutches until review at 6 weeks. I: 35 minute class 2x per week for 6 weeks, no additional home exercises.	12 weeks postoperatively	6	WOMAC, VAS, 6MWT, SF-12, hip abduction strength	18 weeks	I: with better improvement in 6MWT, WOMAC function, and SF-12 Physical. No significant differences in WOMAC pain or stiffness, SF-12 mental health score, VAS, or hip abduction strength
Okoro et al. [19]	C: unsupervised home exercises. I: weekly PT sessions for 6 weeks.	Upon discharge	6	Maximum voluntary contraction of operated leg quad, STS, TUG, stair climb, 6MWT, lean mass of operative leg (DEXA)	6 weeks, 6 months, 9-12 months	No significant difference in any of measured outcomes
Total			8^a^			

Data synthesis and meta-analysis

Summaries for all outcome assessments are summarized in Table 3. No meta-analysis was conducted for long-term aerobic capacity as there were fewer than three studies with available data.

**Table 3:** GRADE assessment of meta-analytic results. GRADE: Grading of Recommendations Assessment, Development and Evaluation, I^2^: I-square heterogeneity statistic, LE: lower extremity.

Outcome	Risk of bias	Directness of evidence	Heterogeneity	Precision	Publication bias	Overall quality
LE strength short term	No downgrade	No downgrade. No evidence of indirectness.	I^2^ = 66%, moderate heterogeneity. Downgraded one level	Rated down one level. Moderate imprecision.	No assessment of publication bias conducted.	Low
LE strength long term	Downgraded by one level. Limitation primarily in selection of reported result.	No downgrade. No evidence of indirectness.	I^2^ = 22%, low heterogeneity	Rated down one level. Moderate imprecision.	No assessment of publication bias conducted.	Low
Aerobic capacity short term	No downgrade	No downgrade. No evidence of indirectness.	I^2^ = 12%, low heterogeneity	Rated down two levels. Significant imprecision. Very wide confidence interval. Well underneath suggested sample size.	No assessment of publication bias conducted.	Low
Self-reported physical outcome short term	Downgraded by one level. Limitation primarily in measurement of outcome.	No downgrade. No evidence of indirectness.	No downgrade. I^2^ = 0%, low heterogeneity	Rated down one level. Moderate imprecision.	No assessment of publication bias conducted.	Low
Self-reported physical outcome long term	Downgraded by one level. Limitation primarily in measurement of outcome.	No downgrade. No evidence of indirectness.	I^2^ = 0%, low heterogeneity	Rated down one level. Moderate imprecision.	No assessment of publication bias conducted.	Low
Self-reported QoL short term	Downgraded by one level. Limitation primarily in measurement of outcome.	No downgrade. No evidence of indirectness.	I^2^ = 0%, low heterogeneity	Rated down one level. Moderate imprecision.	No assessment of publication bias conducted.	Low
Self-reported QoL long term	Downgraded by one level. Limitation primarily in measurement of outcome.	No downgrade. No evidence of indirectness.	I^2^ = 0%, low heterogeneity	Rated down one level. Moderate imprecision.	No assessment of publication bias conducted.	Low

Short-Term Outcomes

**Figure 2:**
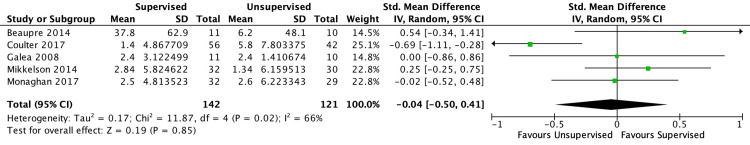
Forest plot for five randomized controlled trials investigating short-term lower extremity strength in unsupervised vs. supervised exercise regimens following primary total hip arthroplasty. References: Beaupre et al. [8]; Coulter et al. [16]; Galea et al. [15]; Mikkelson et al. [17]; Monaghan et al. [18].

**Figure 3:**
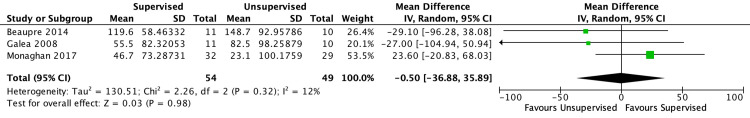
Forest plot for three randomized controlled trials investigating short-term aerobic capacity in unsupervised vs. supervised exercise regimens following primary total hip arthroplasty. References: Beaupre et al. [8]; Galea et al. [15]; Monaghan et al. [18].

**Figure 4:**
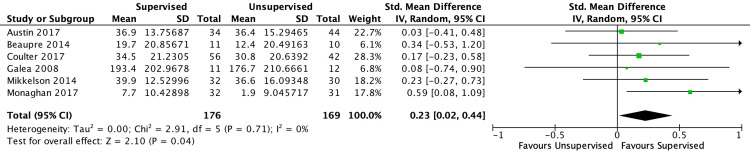
Forest plot for six randomized controlled trials investigating short-term patient-reported physical outcome scores in unsupervised vs. supervised exercise regimens following primary total hip arthroplasty. References: Austin et al. [7]; Beaupre et al. [8]; Coulter et al. [16]; Galea et al. [15]; Mikkelson et al. [17]; Monaghan et al. [18].

**Figure 5:**
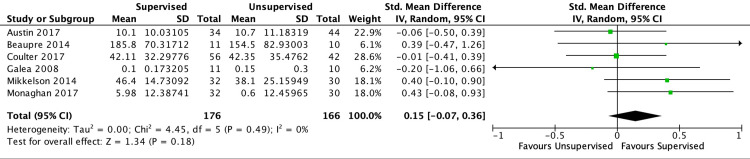
Forest plot for six randomized controlled trials investigating short-term patient-reported quality of life scores in unsupervised vs. supervised exercise regimens following primary total hip arthroplasty. References: Austin et al. [7]; Beaupre et al. [8]; Coulter et al. [16]; Galea et al. [15]; Mikkelson et al. [17]; Monaghan et al. [18].

Of the six studies included in the short-term outcome analysis, five found both interventions to be equivocal. One study found a statistically significant difference in physical function scores favoring the supervised cohort but was unable to determine if this difference was clinically significant. Based on five studies and a low level of certainty, no differences in short-term LES were found (SMD −0.04 [−0.50, 0.41]; I2=66%; p=0.85) (Figure 2). There was no significant difference in short-term aerobic capacity based on three studies and a low level of certainty (SMD −0.50 [−36.88, 35.89]; I2=12%; p=0.98) (Figure 3). Compared with unsupervised home exercise, the supervised exercise regimen was associated with improved self-reported physical function outcome scores based on six studies and a low level of certainty (SMD 0.23 [95% CI, 0.02-0.44]; I2=0%; p=0.04) (Figure 4). According to Cohen's work [20,21], an SMD of 0.23 is considered a small effect size. No differences were found between the two cohorts with regard to short-term QoL scores based on six studies and a low level of certainty (SMD 0.15 [−0.07, 0.36]; I2=0%; p=0.18) (Figure 5).

Long-Term Outcomes

**Figure 6:**
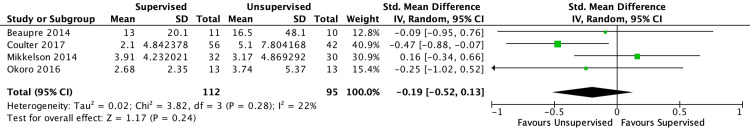
Forest plot for four randomized controlled trials investigating long-term lower extremity strength in unsupervised vs. supervised exercise regimens following primary total hip arthroplasty. References: Beaupre et al. [8]; Coulter et al. [16]; Mikkelson et al. [17]; Okoro et al. [19].

**Figure 7:**
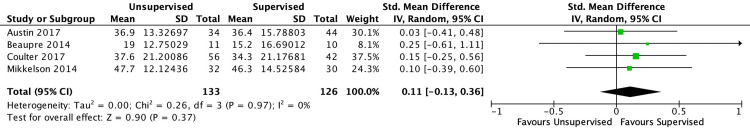
Forest plot for four randomized controlled trials investigating long-term patient-reported physical outcome scores in unsupervised vs. supervised exercise regimens following primary total hip arthroplasty. References: Austin et al. [7]; Beaupre et al. [8]; Coulter et al. [16]; Mikkelson et al. [17].

**Figure 8:**
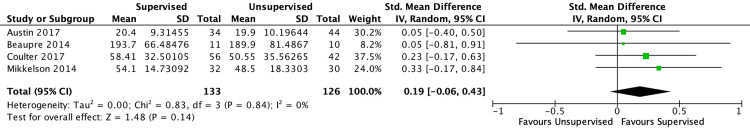
Forest plot for four randomized controlled trials investigating long-term patient-reported quality of life scores in unsupervised vs. supervised exercise regimens following primary total hip arthroplasty. References: Austin et al. [7]; Beaupre et al. [8]; Coulter et al. [16]; Mikkelson et al. [17].

No long-term outcome differences were identified between the unsupervised and supervised cohorts in this study. There was no significant difference observed with regards to long-term LES, based on four studies and a low level of certainty (SMD −0.19 [−0.52, 0.13]; I2=22%; p=0.24) (Figure 6). Similarly, no differences were observed with regards to long-term patient-reported physical outcome scores, based on four studies and a low level of certainty (SMD 0.11 [−0.13, 0.36]; I2=0%; p=0.37) (Figure 7), or long-term QoL scores based on four studies and a low level of certainty (SMD 0.19 [−0.06, 0.43]; I2=0%; p=0.14) (Figure 8).

Risk of bias

The results of the quality appraisal are summarized in risk of bias summary plots in the appendices (Appendix Figures 9-15). The self-reported scores all had a high risk of bias, primarily due to bias in outcome measurement (Table 3). All outcomes were rated as low-quality evidence (Table 4). The primary reasons for the downgrade in quality were the risk of bias and imprecision. Publication bias was not assessed as there were fewer than 10 studies involved in each outcome.

**Table 4:** Summary of findings table. SMD: standardized mean difference, MD: mean difference CI: confidence interval, QoL: quality of life, LE: lower extremity. ^a^Standardized mean difference. ^b^Mean difference.

Outcome	Supervised participants (n)	Unsupervised participants (n)	SMD/MD (95% CI)	Risk of bias	Certainty of evidence
LE strength short term	142 (5)	121 (5)	−0.04 (−0.50, 0.41)^a^	Some concerns	Low ⨁⨁◯◯
LE strength long term	112 (4)	95 (4)	−0.19 (−0.52, 0.13)^a^	High	Low ⨁⨁◯◯
Aerobic capacity short term	54 (3)	49 (3)	−0.50 (−36.88, 35.89)^b^	Some concerns	Low ⨁⨁◯◯
Self-reported physical outcome short term	176 (6)	169 (6)	0.23 (0.02, 0.44)^ a^	High	Low ⨁⨁◯◯
Self-reported physical outcome long term	133 (4)	126 (4)	0.11 (−0.13, 0.36)^a^	High	Low ⨁⨁◯◯
Self-reported QoL short term	176 (6)	166 (6)	0.15 (−0.07, 0.36)^a^	High	Low ⨁⨁◯◯
Self-reported QoL long term	133 (4)	126 (4)	0.19 (−0.06, 0.43)^a ^	High	Low ⨁⨁◯◯

Discussion

Despite the historical emphasis on the importance of formal PT as a critical intervention after THA, this meta-analysis fails to demonstrate any benefit for PT over unsupervised home exercises aside from a small increase in short-term self-reported physical function scores. No significant differences were found with regards to short- and long-term changes from baseline for LES, aerobic capacity, and self-reported QoL scores, as well as long-term self-reported physical outcome scores. The results of our meta-analysis suggest that arthroplasty providers should question the routine use of formal PT for all primary THA patients.

Other reviews conducted on formal PT programs following THA provide mixed results. Reviews conducted by Lowe et. al. [22] and Wijnen et. al. [23] did not perform a meta-analysis of physical function due to considerable variation in their included studies and were unable to provide a definitive conclusion; however, the latter reported an association with increased hip abductor muscle strength. A review conducted by Fatoye et al. [24] including RCTs and retrospective cohorts found that formal PT improved both physical function scores and hip abduction strength, but did not differentiate between short- or long-term follow-up points (follow-up ranged from 2 weeks to 12 months). Finally, Sauressig et al. [25] similarly conducted a meta-analysis and found no differences in self-reported physical function at 4 weeks, 12 weeks, 26 weeks, and one year.

An important aspect of the current review separating it from these prior studies is the use of a clearly defined home exercise regimen, postoperative instructions, or a booklet on discharge as inclusion criteria, excluding those studies that did not specify any form of intervention for their control groups. This is important to ensure a low-cost, standardized control group to allow providers to understand the true effect of trained therapist-led PT for their patients. Without this aspect, studies with controls consisting of no intervention could potentially be included in this review, which could bias results toward the formal therapy groups and may not reflect the current state of most practices.

Although postoperative rehabilitation has long been linked to a successful outcome following THA, the use of supervised PT has several drawbacks. Copay affordability, scheduling outpatient appointments, and arranging transportation have been demonstrated to be legitimate barriers to accessibility to outpatient PT for THA patients after discharge [10,11]. PT exercises can also be painful, as one of the most commonly asked questions regarding PT in the postoperative period after THA is about pain expectations [26]. Additionally, Yayac et al. demonstrated that TJA patients who underwent supervised PT had a significantly higher readmission rate than those who were discharged with self-directed home exercise regimens [9]. After controlling for patient demographics and comorbidities, they found that patients who had supervised home PT were over three times more likely to be readmitted in the 90-day postoperative period. Finally, the added cost associated with it must be considered. Yayac et al. [9] analyzed costs and outcomes in their retrospective study; while no clinically significant difference was found between function or quality of life between groups at two years, they concluded that formal therapy costs included 8% of a 90-day episode of care costs for those receiving supervised home PT and outpatient PT and 3% for those receiving supervised home PT only [9]. The results of the current study highlight the need to re-examine the application of routine PT following primary THA, especially with post-discharge costs accounting for up to 36% of total costs in the bundle payments for TJA [27]. This is particularly salient in the context of a younger patient population, improvements in pain management, and emphasis on early mobilization postoperatively.

The difference between the short-term, self-reported physical outcome scores we observed between supervised and unsupervised groups was largely driven by the findings of the study conducted by Monaghan et al. [18]. This RCT involving an exercise intervention consisting of land- and aquatic-based therapy performed between 12 and 18 weeks after surgery differed from the other studies in this review as it was the only one to include an aquatic component. Additionally, while they found a statistically significant relationship between their formal exercise intervention and improved Western Ontario and McMaster Universities Arthritis Index (WOMAC) scores, they were unable to state whether this translated to a clinically significant difference - an issue that has been raised with the use of WOMAC scores in other literature [28,29]. Our meta-analysis reported an effect size of 0.23, falling under the category of small effect size as described by Cohen [20,21]. However, this was the only measure demonstrating a difference between the two interventions with regard to short-term outcomes and was likely driven by a single study. In the three studies that analyzed data at 12 months, no significant differences were found in any of the assessed outcomes between the two groups [7,8,19]. At the six-month time point, Mikkelson et al. [17] found a larger increase in maximal walking speed and stair climb performance in the formal therapy group, while Johnsson et al. [30] reported increased intermediate-to-moderate pain at six months in the home exercise group. Two other studies at the six-month mark found no significant differences in physical or mental outcomes between home exercise and formal PT groups.

Our results are limited by the number of studies and the quality of their respective data included in the meta-analysis. An important aspect to consider is the included patient population of each included study. Eligibility criteria may have preselected healthier and more motivated patients. In light of this, deconditioned patients with substantial functional deficits or medical comorbidities may still benefit from supervised PT. Additionally, there was substantial heterogeneity of exercise regimens across studies, including aspects such as methodology and duration of therapy, reflecting the likely variation in PT programs at different institutions. Furthermore, relying on patient-reported outcomes can be flawed, particularly in RCTs in which patients know they will be assigned to either supervised or unsupervised cohorts. It is likely that many patients in the intervention groups may be subject to bias - their assignment to formal PT protocols may influence and potentially inflate the supervised PT cohort self-reported outcome scores. The measurement of LES was also subject to variability as the studies assessing it used different methodologies to determine it. Additionally, within the unsupervised cohorts, we were unable to assess the degree of compliance; however, the intention-to-treat approach taken by most of these studies is likely to replicate true scenarios. Finally, previous studies have noted an 18-31% cross-over rate from self-directed exercise to formal supervised therapy in total joint arthroplasty populations [7,31]. We were unable to account for patients who required crossover in our study - outcomes for these patients would be an area of interest in future studies. The strengths of this study include the narrow inclusion criteria, such as the inclusion of only RCTs or that the unsupervised cohorts must have clearly delineated instructions or booklets given to them, to allow for stronger conclusions than previous reviews on this subject.

## Conclusions

Despite a historical emphasis on the importance of formal PT after primary THA, the currently available literature fails to demonstrate a significant benefit for formal PT over unsupervised home exercise. However, this does not include the possibility that certain subgroups of deconditioned patients may be aided by supervised therapy. Our results may help providers educate their patients on whether to pursue formal PT programs postoperatively. With little demonstrated benefit aside from a short-term increase in self-reported physical outcome scores, this review suggests that the routine use of formal PT may not warrant its cost.

## Figures and Tables

**Figure 9:**
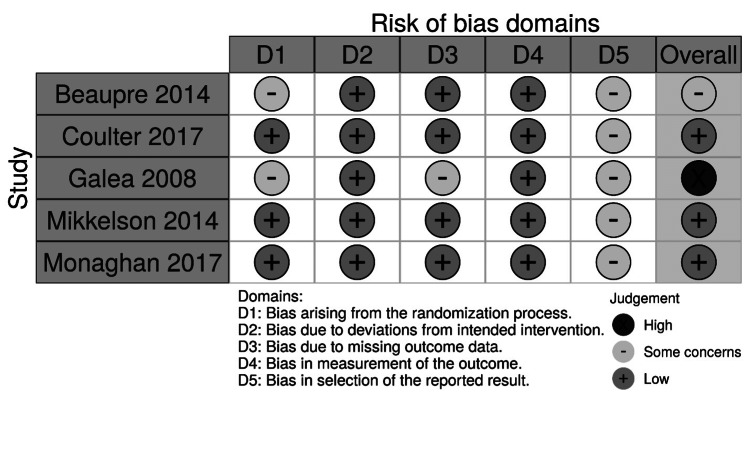
Risk of bias summary chart for short-term change in lower extremity strength in five randomized controlled trials. References: Beaupre et al. [8]; Coulter et al. [16]; Galea et al. [15]; Mikkelson et al. [17]; Monaghan 2017 [18].

**Figure 10:**
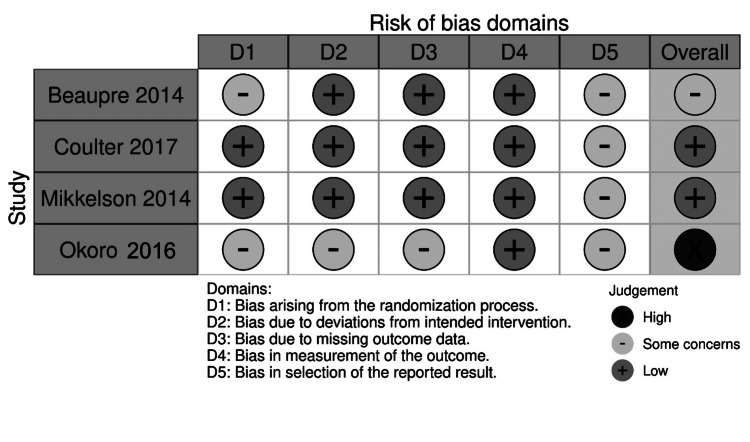
Risk of bias summary chart for long-term change in lower extremity strength in four randomized controlled trials. References: Beaupre et al. [8]; Coulter et al. [16]; Mikkelson et al. [17]; Okoro et al. [19].

**Figure 11:**
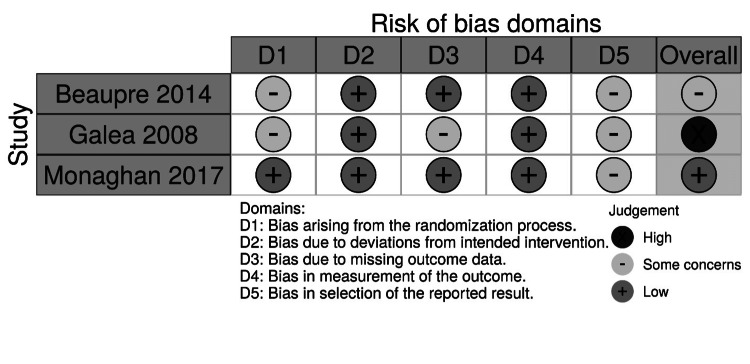
Risk of bias summary chart for short-term change in aerobic capacity in three randomized controlled trials. References: Beaupre et al. [8]; Galea et al. [15]; Monaghan et al. [18].

**Figure 12:**
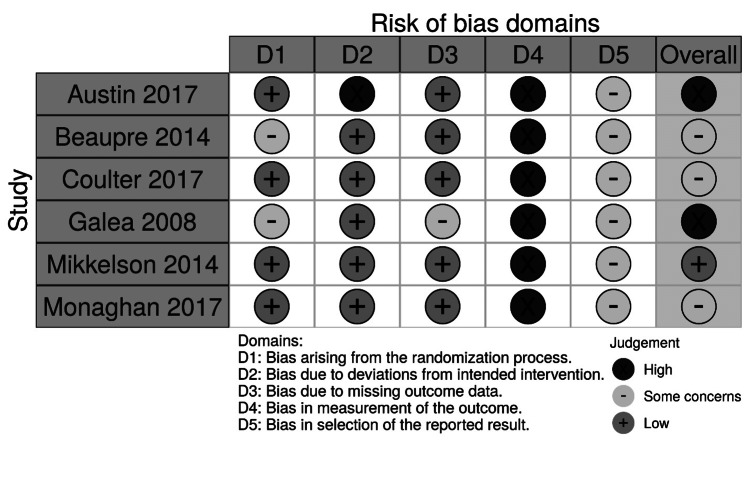
Risk of bias summary chart for short-term change in patient-reported physical function in six randomized controlled trials. References: Austin et al. [7]; Beaupre et al. [8]; Coulter et al. [16]; Galea et al. [15]; Mikkelson et al. [17]; Monaghan et al. [18].

**Figure 13:**
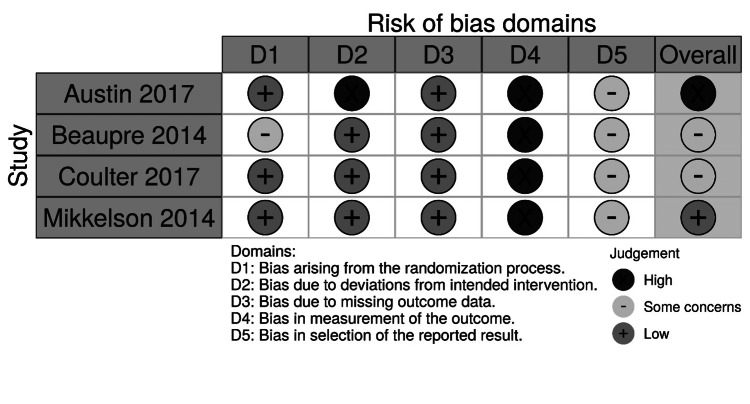
Risk of bias summary chart for long-term change in patient-reported physical function in four randomized controlled trials. References: Austin et al. [7]; Beaupre et al. [8]; Coulter et al. [16]; Mikkelson et al. [17].

**Figure 14:**
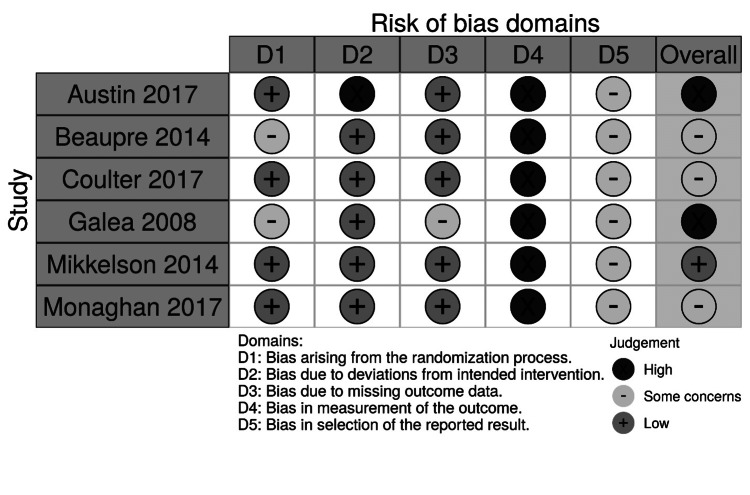
Risk of bias summary chart for short-term change in patient-reported quality of life in six randomized controlled trials. References: Austin et al. [7]; Beaupre et al. [8]; Coulter et al. [16]; Galea et al. [15]; Mikkelson et al. [17]; Monaghan et al. [18].

**Figure 15:**
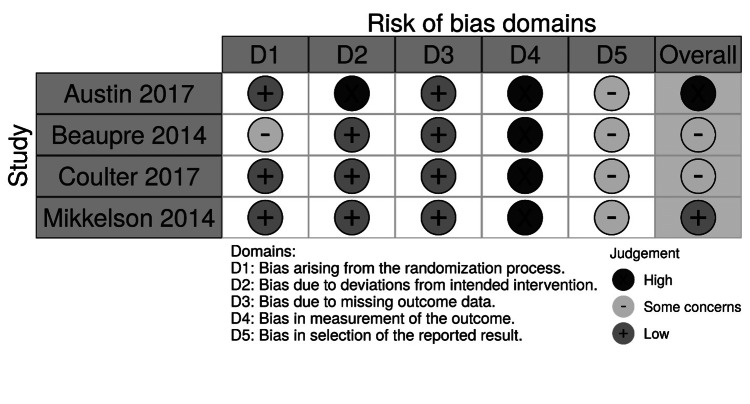
Risk of bias summary chart for long-term change in patient-reported quality of life in six randomized controlled trials. References: Austin et al. [7]; Beaupre et al. [8]; Coulter et al. [16]; Mikkelson et al. [17].
